# Evaluation of Potency and Duration of Immunity Elicited by a Multivalent FMD Vaccine for Use in South Africa

**DOI:** 10.3389/fvets.2021.750223

**Published:** 2021-12-15

**Authors:** Faith R. M. Peta, M. M. Sirdar, Peter van Bavel, P. B. Mutowembwa, N. Visser, J. Olowoyo, M. Seheri, Livio Heath

**Affiliations:** ^1^Transboundary Animal Diseases: Vaccine Production Programme, Onderstepoort Veterinary Research Institute, Agricultural Research Council, Pretoria, South Africa; ^2^Department of Medical Virology, School of Medicine, Sefako Makgatho Health Sciences University, Pretoria, South Africa; ^3^Private Consultants, Boxmeer, Netherlands; ^4^Department of Biology, School of Science and Technology, Sefako Makgatho Health Sciences University, Pretoria, South Africa

**Keywords:** vaccine candidate, 50% protective dose (potency), duration of immunity, humoral immunity, clinical protection, South Africa

## Abstract

South Africa (SA) experiences sporadic foot and mouth disease (FMD) outbreaks irrespective of routine prophylactic vaccinations of cattle using imported commercial vaccines. The problem could be mitigated by preparation of vaccines from local virus strains related to those circulating in the endemically infected buffalo populations in the Kruger National Park (KNP). This study demonstrates the individual number of protective doses (PD) of five vaccine candidate strains after homologous virus challenge, as well as the vaccines safety and onset of humoral immunity in naïve cattle. Furthermore, the duration of post-vaccination immunity over a 12-month period is shown, when a multivalent vaccine prepared from the five strains is administered as a primary dose with or without booster vaccinations. The five monovalent vaccines were shown to contain a 50% PD between 4 and 32, elicit humoral immunity with antibody titers ≥2.0 log10 from day 7 post-vaccination, and cause no adverse reactions. Meanwhile, the multivalent vaccine elicited antibody titers ≥2.0 log10 and clinical protection up to 12 months when one or two booster vaccinations were administered within 6 months of the primary vaccination. An insignificant difference between the application of one or two booster vaccinations was revealed. Owing to the number of PDs, we anticipate that the multivalent vaccine could be used successfully for prophylactic and emergency vaccinations without adjustment of the antigen payloads. Furthermore, a prophylactic vaccination regimen comprising primary vaccination of naïve cattle followed by two booster vaccinations 1.5 and 6 months later could potentially maintain herd immunity over a period of 12 months.

## Introduction

Foot and mouth disease (FMD) is a highly infectious and economically important disease of cloven-hoofed animals (1). The disease is caused by the FMD virus (FMDV), an *Aphthovirus* of the *Picornaviridae* family (2), and it is characterized by fever of up 42°C during the viremic stages of infection and lesions in the mouth, especially on the tongue, gums, and hooves (3, 4). The virus occurs in seven antigenically and genetically distinct serotypes, namely, A, C, O, Asia-1, and Southern African Territories (SAT) 1, 2, and 3 (5, 6), characterized by lack of cross-protection among and within serotypes (7). All three SAT serotypes are endemic in the African buffalo populations in most African countries, including the Southern African Development Community (SADC) region, and therefore pose the continual threat of infecting livestock herds at wildlife–livestock interfaces (8, 9).

Owing to the global FMD regulations, many parts of Africa, Asia, and the Middle East endemically affected by the disease experience economic barriers, such as inability to trade in livestock and livestock products (10). Because most developed countries are FMD-free, the economic ramifications of FMD are mostly felt in developing countries (11). Vaccination of livestock is an integral component of FMD control worldwide, and billions of vaccine doses are used each year in disease control or eradication campaigns (12–14). To enable accesses to livestock and livestock products markets, some countries such as South Africa (SA) and neighboring countries within SADC have created FMD-free zones without vaccination wherein regional and international trade in livestock and livestock products is allowed and practiced (15, 16). To maintain this FMD-free status, meticulous prophylactic vaccinations of livestock and strict zoo sanitary measures are stringently practiced in areas adjacent to endemically infected zones (6).

Foot and mouth disease control methods differ from country to country, depending on the country's FMD status and available resources (16). In the 1980s, effective vaccines were used successfully to eradicate FMD from continental Europe (17). However, eradication through vaccination is impossible in most African countries owing to the presence of African buffalo populations in game reserves, which are the maintenance hosts of the SAT serotypes (10). Therefore, routine prophylactic vaccination of livestock in high-risk areas (protection zones), in combination with other strategies such as animal movement restrictions and surveillance, are relied upon for the control of disease and maintenance of FMD-free zones without vaccination (6, 15, 16). Successful vaccination regimens, be it for eradication campaigns, the elimination of circulating virus during outbreaks, prophylaxis, maintenance of freedom from the disease, or regaining freedom from the disease, require the use of high quality and efficacious vaccines (18).

Most FMD vaccines are formulations of one or more chemically inactivated viruses derived from cell cultures and blended with suitable adjuvant and excipients (19). Post-vaccination protection against infection is subsequently achieved by the induction of high levels of antibodies (20). Owing to the lack of long-lasting protective immunity after vaccination with SAT serotypes vaccines, repeated (booster) vaccination regimens are practiced in SADC to maintain high levels of antibody titers post-vaccination (21).

Factors such as potency, antigenic relatedness of vaccine strains and the circulating field strains, and structural integrity of the virus; 146 S particle can influence the vaccine's ability to induce protective immunity (22). A vaccine's potency is a measure of the number of protective doses (PD) in a vaccine estimated from the resistance to infectious virus challenge of animal groups immunized with different amounts of a vaccine. One 50% protective dose (PD50) represents the dose of vaccine that will protect 50% of vaccinated animals against clinical FMD (19). Also playing a crucial role in vaccine potency is the antigen payload (19, 23) and the type of adjuvant used for vaccine preparation, which could have an effect on the duration of immunity (24, 25). Commonly used adjuvants for the preparation of FMD vaccines are aluminum hydroxide together with saponin (26, 27) and oil emulsion adjuvants such as the Montanide™ ISA 206 VG [double oil emulsion (DOE)] adjuvant (25). The use of Montanide™ DOE-adjuvanted FMD vaccines in cattle have been shown to elicit higher and longer-lasting antibody titers, although their efficacy can be influenced by the formulation recipe (27–29).

In SA, cattle herds surrounding the Kruger National Park (KNP) game reserve are currently vaccinated up to four times per year using imported commercial vaccines, prepared from cell culture-derived chemically inactivated SAT 1, 2, and 3 antigens, adjuvanted with aluminum hydroxide and saponin (30). However, there is evidence that these vaccines perform poorly, shown by recurrent and persistent outbreaks in vaccinated populations even after increased frequency of vaccinations (31). Poor field performance of the vaccine batches used could be caused by several factors including the disintegration of the virus' 146 S particle during antigen preparation for vaccine formulation, owing to the labile nature of the SAT serotypes (1, 9). However, owing to the quality clearance through quality control (QC) test results appearing on the certificate of analysis of the vaccine batches used, poor field performance of the vaccines was more likely to be a result of antigenic variation between the vaccine strains and the circulating field strains in SA, since the vaccine strains as well as its potency were not disclosed.

In this study, Nguni cattle were used to evaluate the potency of five monovalent vaccines prepared with virus strains representing the three local SAT serotypes circulating in the FMD control zone of SA and Montanide™ ISA 206 VG adjuvant. Thereafter, the duration of immunity in cattle elicited by the five strains as a multivalent vaccine was investigated after administration of primary vaccination with and without booster vaccinations.

## Materials and Methods

### Preparation of Vaccines and Challenge Viruses

Vaccine candidate strains were supplied by the in-house Office International des Epizooties (OIE) reference laboratory for FMD, and they were a selection of five virus isolates representing the three SAT serotypes that had previously caused outbreaks within the FMD control zone in SA. The five virus strains, namely, SAT 1: SAR 9/81 and BOT 1/06, SAT 2: KNP1/10 and SAR 3/04, and SAT 3: KNP 10/90, were in addition shown to be antigenically related with heterologous reference field strains (*r*_1_-values ≥0.3). Their molecular epidemiological relationships, determined by VP1 sequencing and phylogenetic analysis conducted by the same laboratory, revealed that they were genetically related to other isolates of respective serotypes from topotype I (Mozambique, SA, and Zimbabwe).

The viruses were prepared in an in-house modification of Baby Hamster Kidney (BHK)-21 clone 13 (ATCC CCL-10) suspension cell cultures as previously described (32). Inactivation was conducted according to Bahnemann (33), for 24 h at 35°C using two doses of 2.5 mM Binary Ethyleneimine (BEI, Merck, Darmstadt, Germany) each, at the beginning of inactivation (0 h) and after 4 h when the culture was transferred to the second inactivation vessels. Antigens were clarified by centrifugation at 1,000 rpm for 10 min at 25°C and collected as supernatants before they were transferred to the second inactivation vessel. Concentration and purification of the antigens was conducted by precipitation in polyethylene glycol 6000 (Merck, Darmstadt, Germany) as previously described (21). Subsequently, antigens equivalent to 200 vaccine doses were tested for innocuity in BHK monolayer cells as described in the OIE terrestrial manual (19).

Five monovalent vaccines comprising antigen payloads of 3.0 μg/ml for SAT 1 and SAT 3 strains and 6.0 μg/ml for SAT 2 strains were successively prepared combined with other vaccine excipients to form the aqueous phase of the vaccines. The vaccines' aqueous phase was mixed 1:1 (v/w) with Montanide™ ISA 206 VG oil adjuvant according to the manufacturer's guidelines for vaccine formulation (34), and the vaccines were, respectively, named SAT 1A, SAT 1B, SAT 2A, SAT 2B, and SAT 3 vaccines.

Similarly, all five strains comprising the antigen payloads mentioned above were combined and used as a multivalent vaccine, to evaluate the duration of immunity over a period of 12 months. All vaccines were subsequently stored at 4°C, while a sample of each was subjected to the sterility (19, 35) and 146 S particle (36, 37) QC tests.

#### Animal Ethics Clearance, Sourcing, and Handling

The study was conducted in line with the animal welfare and ethics guidelines of the Agricultural Research Council-Onderstepoort Veterinary Research Institute (ARC-OVI) Animal Ethics Committee (AEC) and was approved under study reference number 12/11/1/1a. Furthermore, the national Department of Agriculture, Land Reform and Rural Development (DALRRD) provided clearance in accordance with Section 20 of the Animal Diseases Act, Act No. 35 of 1984.

A total of 160 Nguni cattle (males and females 6 months of age) were sourced from an FMD-free zone and quarantined in open pens at the OVI large animal farm. Animals were bled from the jugular vein in vacutainer blood collection tubes, and the serum samples collected from clotted blood were tested for pre-vaccination (day 0) FMD antibody titers.

Ten days before vaccination or inoculation with FMDV, animals were admitted per study group to biosafety level 3 (BSL-3) animal holding facility of the OVI-Transboundary Animal Diseases Laboratory and housed in self-contained stables with a minimum floor surface of at least 25.23 m^2^. The cattle were able to move freely within the confines of the stables. Animals were fed a balanced commercial ration of high-roughage pellets once a day, water was provided *ad libitum* by the automated watering system, and the stables and food troughs were cleaned daily. The environment within the stables was controlled at relative humidity of ±35% and temperature of ±23°C. The pressure was maintained at approximately −40 Pa, and light was ±600 lux (automatically switched on around 06:00 a.m. and off around 6:00 p.m.).

#### Preparation of Homologous Viruses for Post-vaccination Challenge Studies

Five virus isolates (SAR 9/81/1; BOT 1/06/1; KNP 1/10/2; SAR 3/04/2, and KNP10/90/3) homologous to the vaccine strains were received as cell culture-based virus suspensions of known infective titers [50% Tissue culture infectious dose (TCID50)/ml], with passage history of primary pig kidney_1_, Instituto Biologico Renal Suino-2_2_, and baby hamster kidney_5_ cells (PK_1_IB-RS2_2_BHK_5_), from the in-house OIE reference laboratory for FMD. The virus cultures were diluted to a final concentration of 10^4^ TCID50, and 0.2 ml (2,000 TCID50) of each virus suspension was inoculated intra-dermolingually in two Nguni cattle per strain, following sedation with 0.22 mg/kg xylazine (Bayer, Germany). Bovine adapted challenge viruses were collected from resultant epithelial lesions of the tongues and feet (hooves) 48 h post-inoculation and used as challenge viruses.

### Monovalent Vaccines PD50 (Potency) Evaluation

The PD50 evaluation was conducted according to the OIE Terrestrial manual (38). Briefly, 17 animals per monovalent vaccine potency evaluation were admitted to the BSL-3 animal holding facility and assigned identity numbers with ear tags and randomly divided into three vaccination groups and an unvaccinated control group of two animals. The three vaccination groups were vaccinated intramuscularly with reducing vaccine volumes per monovalent vaccine (Table 1).

**Table 1:** Cattle grouping and administration of monovalent vaccines for PD50 (potency) test.

	**Test groups**			**Controls**
**Monovalent vaccines names**	**Group 1** **Full** **(2 ml)**	**Group 2** **1/4** **(0.5 ml)**	**Group 3** **1/16** **(0.125 ml)**	**Group 4** **unvaccinated** **control**
	**Number of animals per group**			
SAT 1A	5	5	5	2
SAT 1B	5	5	5	2
SAT 2A	5	5	5	2
SAT 2B	5	5	5	2
SAT 3	5	5	5	2

#### Health Monitoring Post-vaccination

The animals' general health, including rectal body temperatures, were monitored daily for 28 days post-vaccination (dpv) to record development of any adverse skin reactions on the injection site of the vaccines and/or febrile condition. Blood samples were collected every 7 days up to 28 dpv as mentioned previously, from animal groups vaccinated with a 2 ml vaccine dose, to evaluate the onset of humoral immune response.

#### Virus Challenge of Animals and Clinical Scoring Post-challenge

On day 28 post-vaccination, all animals including the unvaccinated control group were sedated and challenged as described before with bovine adapted homologous virus. All animals were examined under sedation every 48 h up to 10 days for post-challenge FMD clinical signs. Rectal temperatures were recorded daily, and temperatures ≥40°C were classified as fever.

Clinical signs were scored as follows: 1 for fever, +1 for tongue lesions irrespective of severity, +2 for lesions on one foot (in addition to tongue lesions), and +1 for lesions on each additional foot. Animals could potentially score a maximum of seven points if presenting lesions on all feet, as well as fever and tongue lesions. Animals with a clinical score >2 were considered unprotected, whereas scores ≤ 2 (fever plus tongue lesions) were considered protected. The Reed and Muench (39) method of 50% end-point estimation was used to calculate each vaccine's PD50 value based on the number of protected against unprotected animals post-challenge.

### Evaluation of the Duration of Immunity (Humoral Response and Clinical Protection) Elicited by the Multivalent Vaccine

#### Animal Grouping

A total of 55 cattle were admitted to the BSL-3 animal holding facility. The animals were divided into three vaccination regimen groups, namely, Group 1 (*n* = 25) for a single primary vaccination at day 0, Group 2 (*n* = 20) for primary vaccination plus two booster vaccinations (1.5 and 6 months post-primary vaccination), and Group 3 (*n* = 10) for primary vaccination plus one booster (6 months post-primary vaccination). Owing to stable sizes in the BSL-3 animal holding facility, the animals were housed in seven subgroups (*n* = 5 or 10) while maintaining the three vaccination regimen groups mentioned above. Based on post-vaccination(s) virus challenge schedule, animals were assigned to subgroups labeled A–G and identified by numbers 1–10 per subgroup using ear tags.

#### Vaccination and Samples Collection Post-vaccination

All animals (*n* = 55) were vaccinated intramuscularly with a 2 ml vaccine dose (primary vaccination) and were thereafter divided into the aforementioned groups. After 1.5 months (6 weeks), a first booster vaccination of the same dosage as the primary vaccination was applied to animals in Group 2. Six months after the primary vaccination, a second booster was applied to Group 2 animals, while Group 3 animals received their first booster vaccination. Monthly blood samples were collected from all animals as mentioned before, up to 12 months post-vaccination (mpv).

#### Virus Challenge Post-vaccination(s)

Ten additional animals were admitted to the BSL-3 animal holding facility in a phased manner of two animals per phase, to serve as unvaccinated controls during virus challenge periods of 1.5, 3, 6, 9, and 12 mpv. Animals were challenged as described before with SAR 3/04/2 at each challenge period. Thus, five animals in Group 1 were challenged at 1.5 mpv, and thereafter, five animals each from Groups 1 and 2 were challenged at 3, 6, 9, and 12 mpv. Similarly, five animals from Group 3 were challenged at 9 and 12 mpv, after receiving their first booster at 6 mpv. Post-challenge animal monitoring was conducted for 10 days as described before, and the animals were terminated thereafter.

#### Serology Testing

##### Onset of Humoral Immunity After Vaccination With Monovalent Vaccines

For evaluation of the onset of humoral immunity, animal groups vaccinated with 2 ml of the five monovalent vaccines were used. The liquid phase blocking enzyme-linked immunosorbent assay (LPBE) was conducted according to Hamblin et al. (40), on serum samples collected pre-vaccination, and every 7 days up to 28 dpv. Antigens homologous to the vaccine strains were used to determine the amount of antibodies (log10 titers) elicited against each monovalent vaccine. Pre-vaccination antibody titers were considered as the baseline humoral immune response, and in accordance with the pre-established trend of the test's negative control titer, serum samples with titers ≥1.6 log10 were considered as indicative of seroconversion to the corresponding serotype (29).

##### Duration of Humoral Immunity After Vaccination With a Multivalent Vaccine

For the evaluation of antibody titers after vaccination with a multivalent vaccine, serum samples collected pre-vaccination and monthly post-vaccination were tested for SAT 2 virus neutralizing (VN) antibody titers in a VN test. The test was conducted as described in the OIE Manual of Diagnostic Tests and Vaccines for Terrestrial Animals (41, 42). Briefly, serially diluted samples as well as known positive and negative controls were incubated with 10^2^ TCID50 of SAR 3/04/2, homologous to SAT 2B vaccine strain for 1 h at 37°C. The mixture was transferred to monolayers of BHK-21clone 13 cells. The end-point titers were calculated as the reciprocal of the last dilution of serum samples to neutralize 100 TCID50 in 50% of the wells after 72-h incubation period in a carbon dioxide incubator at 37°C (43). Pre-vaccination serum titers were recognized as the baseline humoral immune response. Additionally, samples with antibody titers ≤ 1.6 log10 were considered non-neutralizing, in line with the test negative control titer.

#### Statistical Analysis

The normality assumption for all quantitative outcome variables was assessed by calculating descriptive statistics, plotting histograms, and performing Anderson–Darling test for normality. Liquid phase blocking enzyme and VN antibody titers were presented as mean ± standard deviation (SD) at 95% lower and upper confidence intervals, and comparisons were performed using one-way analysis of variance (ANOVA). Virus neutralizing test results were used to compare titers between vaccination groups for the duration of the immunity study by conducting Kruskal–Wallis non-parametric tests at each sampling point post-vaccination. All statistical procedures were performed using IBM SPSS Statistics (Version 27, International Business Machines Corp., Armonk, New York, USA), and results were interpreted at the 5% level of significance.

## Results

### Vaccines QC Tests

All inactivated antigens conformed to statutory inactivation kinetics and recorded no residual live virus in the innocuity test (Table 2). Furthermore, no microbial contamination was recorded in all vaccines (monovalent and multivalent), and thus, they passed the sterility QC test (Table 2). The antigens concentration (146 S particle) in the formulated vaccines were equivalent to those used during vaccine formulations (results not shown).

**Table 2:** A summary QC test results during antigen production for vaccine formulation.

**Test**	**Description of test conducted**	**Compulsory requirements for the tests**	**Results per strain**				
			**SAT 1A**	**SAT 1B**	**SAT 2A**	**SAT 2B**	**SAT 3**
Inactivation kinetics	Virus titration test on inactivation samples (the rate of viral RNA inactivation)	**Correlation coefficient** ***R*** **≤−0.90**	–0.997	–0.978	–0.996	–0.978	–0.900
	Calculated safety titer reached at two-thirds of inactivation time (16 h for a 24-h inactivation time)	**Safety titer** **−7.0 log10**	–10.03	–13.53	–16.15	–13.58	–18.00
	Time to reach safety titer	**Time** **≤16 h**	13.25	10.81	09.70	10.78	09.40
Sterility	Absence of microbial contaminants	**Negative**	Pass	Pass	Pass	Pass	Pass
Innocuity	Absence of residual live virus before vaccine formulation	**Negative**	Pass	Pass	Pass	Pass	Pass

### Safety and the Onset of Humoral Immunity of the Five Monovalent Vaccines

**Figure 1:**
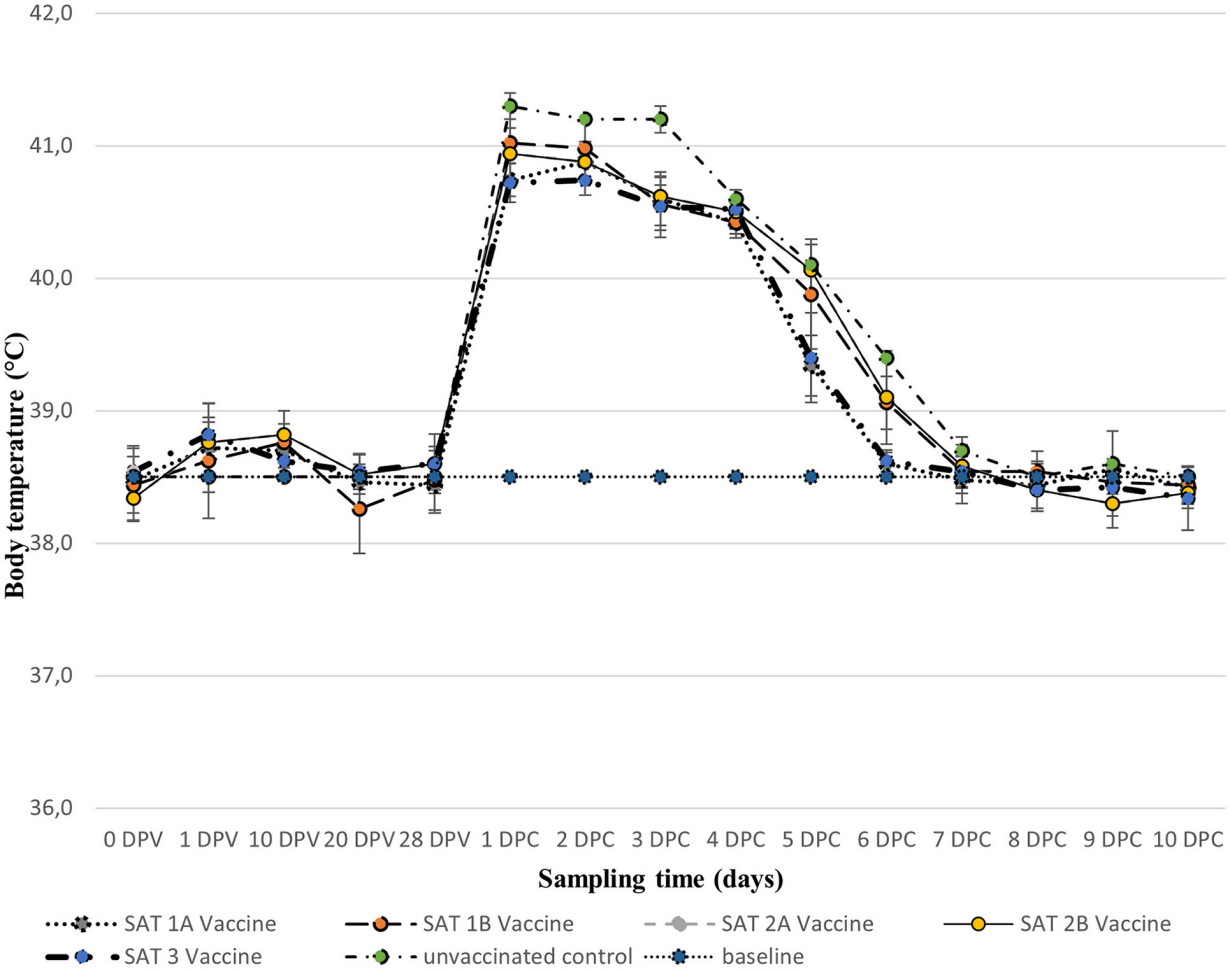
Mean (±SD) rectal body temperatures (°C) of groups of animals vaccinated with 2 ml of the five monovalent vaccines [0–28 days post-vaccination (dpv)] and after respective homologous virus challenge [1–10 days post-challenge (dpc)]. The horizontal dotted line represents the baseline temperature (38.5°C).

No swelling or irritation of the skin was observed at the vaccine's injection site over the 28 dpv with the five vaccines (results not shown). Additionally, no fever was recorded, although a slight increase (<39.0°C) in rectal temperature was recorded in some vaccinated animals across all five vaccines, between 1 and 10 dpv which decreased to approximately 38.5°C by 28 dpv (Figure 1).

**Figure 2:**
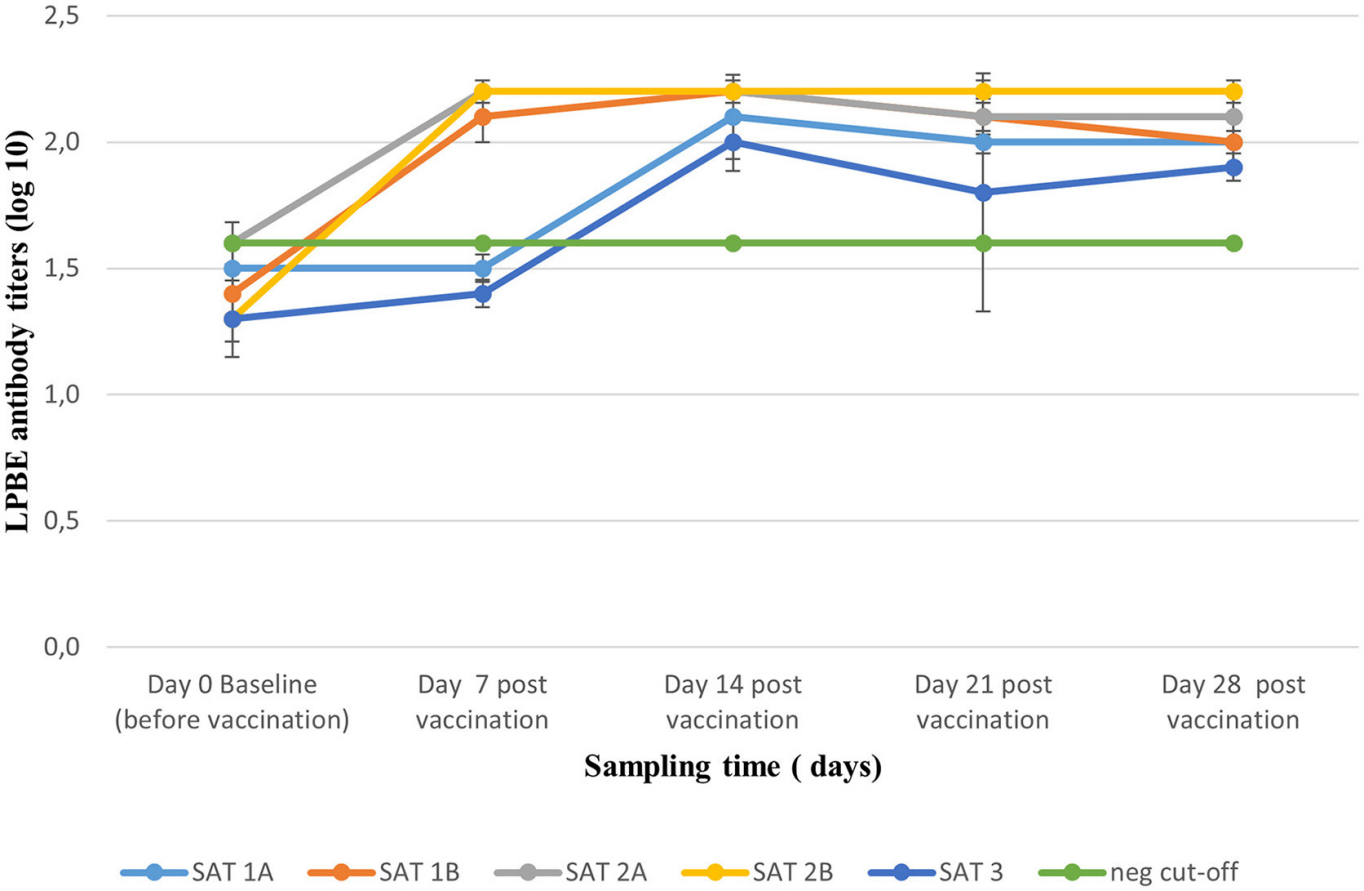
Mean (±SD) antibody titers (liquid phase blocking ELISA) elicited by full-dose vaccination with the five monovalent vaccines, monitored weekly over a period of 28 dpv. The green line represents the cut-off titer of 1.6 log10 based on day 0, pre-vaccination titers.

Homologous LPBE antibody titers elicited by the 2 ml dose of the five vaccines over 28 dpv are presented in Figure 2. All animals recruited including unvaccinated controls reported mean antibody titers ≤ 1.6 log10 before vaccinations (day 0) and were considered FMD negative. Seroconversion was observed from 7 dpv for SAT 1B, SAT 2A, and SAT 2B vaccines when mean (±SD) antibody titers ≥2.0 log10 were recorded and maintained up to 28 dpv. Similar titers were reached from 14 dpv for SAT 1A and SAT 3 vaccines (Figure 2).

#### Monovalent Vaccines PD50 Post-challenge With Homologous Viruses

All animals including unvaccinated controls presented with fever (rectal temperature ≥40.0°C) a day after the challenge, which persisted up to day 3 post-challenge and dropped down to 38.5°C between days 6 and 10 post-challenge (Figure 1).

Classification of animals as clinically protected or unprotected was conducted according to the OIE Manual of Diagnostic Tests and Vaccines for Terrestrial Animals (POTENCY) (2018) (38). Accordingly, animals presenting with feet lesions (clinical scores >2) were considered unprotected. Individual animals' clinical scores were recorded from 1 to 10 days post-challenge (dpc).

In addition to fever, all vaccinated and unvaccinated control animals developed tongue lesions consistent with FMDV infection from 2 dpc (the onset of clinical lesions not shown). Unvaccinated control animals presented with hoof lesions on three or four feet, and accordingly, cumulative clinical scores between 6 and 7 were assigned (Table 3).

**Table 3:**
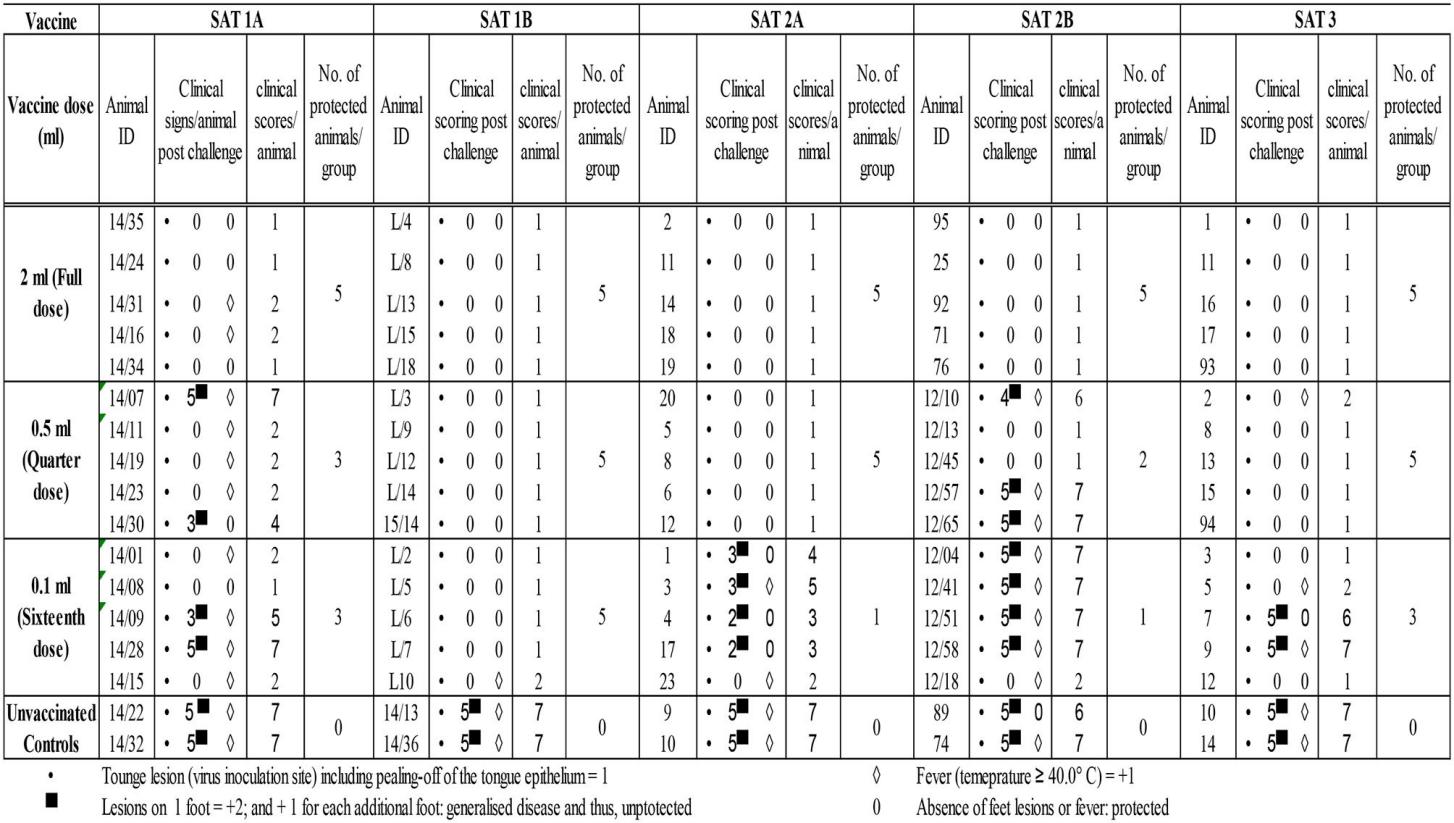
Post-homologous challenge clinical scores for the assessment of vaccines PD50.

The 2 ml vaccine dose across all five vaccines as well as the 0.5-ml dose for SAT 1B, SAT 2A, and SAT 3 vaccines and 0.1-ml dose for SAT 1B vaccines elicited full clinical protection from FMDV post-homologous challenge (clinical score ≤ 2). However, animal protection started to decline with the reduction in vaccination dose administered (Table 3).

Based on the number of protected vs. unprotected animals post-homologous challenge, 12 and 32 PD50 for SAT 1A and SAT 1B vaccines, 10 and 4 for SAT 2A and SAT 2B, and 20 for SAT 3 vaccines were, respectively, assigned (Table 4).

**Table 4:** Monovalent vaccines PD50 post-homologous virus challenge.

	**Vaccination groups**			**PD50**
**Monovalent vaccines**	**Group 1 ** **Full** **(2 ml)**	**Group 2 ** **1/4** **(0.5 ml)**	**Group 3 ** **1/16** **(0.125 ml)**	**Vaccines homologous PD50**
	**Number of protected animals/group**			
SAT 1A	5/5	3/5	3/5	**12**
SAT 1B	5/5	5/5	5/5	**32**
SAT 2A	5/5	5/5	1/5	**10**
SAT 2B	5/5	2/5	1/5	**4**
SAT 3	5/5	5/5	3/5	**20**

*Bold values indicate number of PD50*.

### The Duration of Immunity Elicited by the Multivalent Vaccine

**Figure 3:**
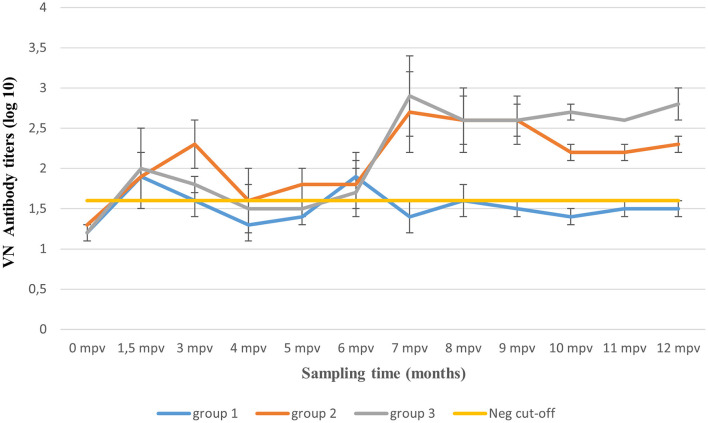
Mean virus neutralizing (VN) antibody titers (±SD) over 12 months post-application of three vaccination regimens. The yellow line represents animals' mean antibody titers (≤ 1.6 log10) before vaccination and the VN test's standard negative control titer.

A total of 15 out of 55 animals across the three vaccination groups died before they could be challenged with a SAT 2 homologous field virus (post-mortem reports not shown) and were therefore excluded from the results. A 12-month overview of clinical results after various challenge periods, and their respective VN antibody titers before challenge inoculations, are illustrated in Table 4, while a monthly report of post-vaccination humoral response between the three vaccination groups is illustrated in Table 5 as well as in Figure 3.

**Table 5:** Duration of immunity after three vaccination groups: VN antibody titers at the time of challenge and clinical results post-challenge.

**Challenge periods**	**1.5 mpv**					**3 mpv**				**6 mpv**					**9 mpv**			**12 mpv**				
**Animal IDs**	**A1**	**A2**	**A3**	**A4**	**A5**	**C6**	**C7**	**C8**	**C9**	**G6**	**G7**	**G8**	**G9**	**G10**	**E 2**	**E 4**	**E5**	**F 1**	**F 2**	**F3**	**F4**	**F5**
**GROUP 1 (SINGLE PRIMARY VACCINATION AT DAY 0)**																						
VN Ab titers	2.3	2.1	2.3	1.7	1.8	2.0	1.8	1.4	1.8	1.6	1.2	1.4	1.6	2.0	1.5	1.5	1.4	1.4	1.4	1.7	1.6	1.6
Clinical results	–	–	–	–	–	–	–	–	+	–	+	–	+	–	–	–	–	–	–	–	–	–
No. protected (% protection)	5/5 (100%)					3/4 (75%)				3/5 (60%)					3/3 (100%)			5/5 (100%)				
**Challenge periods**	**1.5 mpv**					**3 mpv**				**6 mpv**					**9 mpv**			**12 mpv**				
**Animal IDs**						**C1**	**C2**	**C3**	**C5**	**G1**	**G2**	**G3**	**G4**	**G5**	**D1**	**D3**	**D5**	**B1**	**B2**	**B3**		
**GROUP 2 [PRIMARY VACCINATION PLUS TWO BOOSTER VACCINATIONS (1.5 AND 6 mpv)]**																						
VN Ab titers	NC					2.1	2.0	2.2	2.2	2.0	2.0	2.0	1.8	1.2	2.1	2.3	2.6	2.2	2.3	2.4		
Clinical results							–	–	–	–	–	+	–	+	–	–	–	–	–	–	–	
No. protected (% protection)						4/4 (100%)				3/5 (60%)					3/3 (100%)			3/3 (100%)				
**Challenge periods**	**1.5 mpv**					**3 mpv**				**6 mpv**					**9 mpv**			**12 mpv**				
**Animal IDs**																		**D8**	**D9**	**B6**	**B10**	
**GROUP 3 [PRIMARY VACCINATION PLUS ONE BOOSTER VACCINATION (6 mpv)]**																						
VN Ab titers	NC					NC				NC						2.7	2.1	2.9	2.6			
Clinical results																		–	–	–	–	
No. protected (% protection)															2/2 (100%)			2/2 (100%)				

*Ab, antibodies (log10); mpv, months post-vaccination; NC, not challenged; VN, virus neutralizing; –, negative; +, positive*.

#### The Duration of Immunity Over 12 Months, After a Single Primary Vaccination (Group 1)

Before vaccination (0 mpv), all animals recorded mean (±SD) VN antibody titers between 1.2 and 1.3 log10 which was recognized as the baseline humoral response. Based on the titers of the VN test's negative control, a cut-off titer of 1.6 log10 was assigned, and titers >1.6 were recognized as seroconversion. Mean antibody titers of 2.1 log10 were observed before the first five animals were challenged 1.5 mpv (Table 5). A day after the challenge, the five vaccinated animals and the two unvaccinated control animals developed fever (≥40°C) (results not shown). Tongue lesions and subsequent erosion of the tongue epithelia as well as foamy salivation were observed on days 2 up to 5 post-challenge, in the vaccinated animals, while both tongue and feet lesions were observed in two unvaccinated control animals. Thus, 100% clinical protection post-single vaccination was achieved after 1.5 mpv challenge period, as no podal lesions were recorded.

Thereafter, a decline in antibody titers (0.3 log10) was observed, reaching 1.8 log10 (±0.3 SD) by 3 mpv (Figure 3). One vaccinated animal plus the two unvaccinated controls showed both tongue and feet lesions (generalized clinical signs). Seventy-five percent (*n* = 3/4) clinical protection was achieved (Table 5). From 3 to 6 mpv, a further decline in antibody titers was observed, reaching averages between 1.3 and 1.6 log10 (±0.3 SD). Subsequently, 60% (*n* = 3/5) clinical protection was recorded after 6 mpv challenge period (Table 5). Although antibody titers ≤ 1.6 log10 (±0.1 SD) were recorded, 100% clinical protection was achieved after 9 mpv (*n* = 3/3) and 12 mpv (*n* = 5/5) challenge period.

#### The Duration of Immunity Over 12 Months, After Primary Vaccination Plus Two Booster Vaccinations (Group 2)

Animals of this group were boosted at 1.5 mpv and thus not challenged. Mean antibody titers of 2.1 log10 were recorded before 3 mpv challenge challenge, and 100% (*n* = 4/4) clinical protection was achieved (Table 5). However, a waning of antibody titers [up to 1.5 log10 (±0.1 SD)] was recorded between 4 and 5 mpv, irrespective of the 1.5 mpv booster (Figure 3). At 6 mpv, mean antibody titers of 1.8 log10 were recorded (Table 5), and similar to Group 1 animals at the same challenge period, only 60% (*n* = 3/5) clinical protection was achieved. An increase in antibody titers was observed 7 mpv, after administration of the 6 mpv booster vaccination, reaching highs of 2.7 log10 (mean, ±0.3 SD) (Figure 3). In the subsequent months, animals in this group maintained mean antibody titers ≥2.2 log10 up to the end of the study (12 mpv) coinciding with 100% (*n* = 3/3) clinical protection after 9 and 12 mpv challenge (Table 5).

#### Duration of Immunity Over 12 Months, After Primary Vaccination Plus One Booster Vaccination (Group 3)

The 10 animals in Group 3 were only boosted 6 months after the primary vaccination and thus challenged only after 9 and 12 mpv. A total of six animals had died before challenge inoculations. Mean antibody titers of 2.4 log10 (±0.4 SD) and 2.8 log10 (±0.2 SD) (Figure 3) were, respectively, recorded at 9 and 12 mpv corresponding with 100% (*n* = 2/2) clinical protection post-challenge (Table 5). The unvaccinated control groups showed generalized lesions.

#### Statistical (Monthly) Analysis of Post-vaccination Humoral Response of the Three Vaccination Regimes

**Figure 4:**
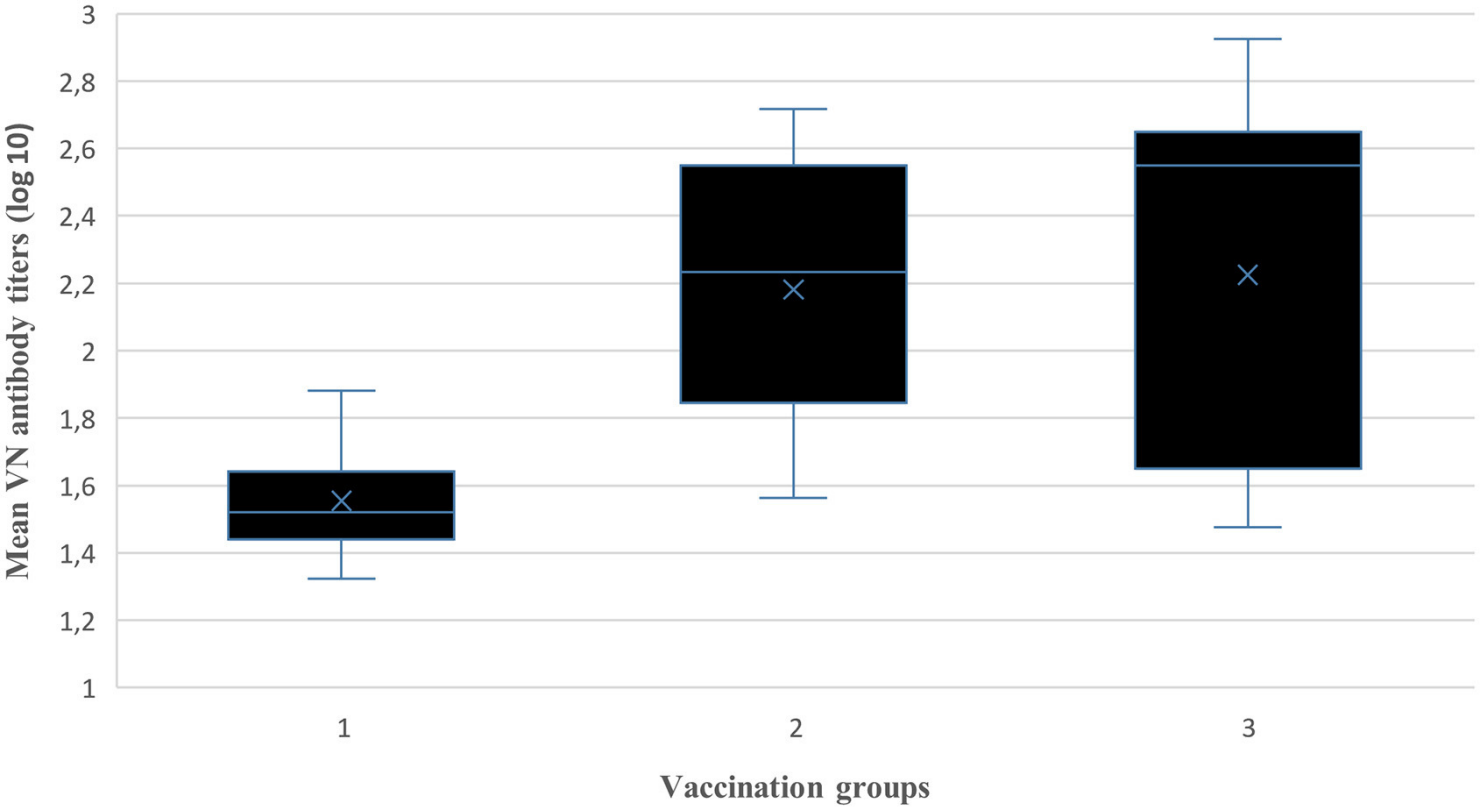
Comparison between vaccination groups of the mean log10 VN titers (independent samples Kruskal–Wallis Test).

Post-vaccination mean antibody titers (±SD) at each sampling interval over 12 months for the three vaccination groups (total animals per group) are presented in Table 6. A reduction in mean antibody titers was observed 3 mpv in animal groups (Groups 1 and 3) without the 1.5 mpv booster vaccination. From 6 to 12 mpv, an increase in antibody titers was observed after administration of the 6 mpv booster vaccination (Groups 2 and 3), whereas titers of Group 1 (without booster) waned sporadically. The ANOVA revealed a difference between the three vaccination groups (*p* < 0.01). The pairwise comparison of groups showed that there is a significant difference between Group 1 and Group 2 (*p* < 0.01) and between Group 1 and Group 3 (*p* < 0.01), while there was no significant difference between Group 2 and Group 3 (*p* > 0.05) (Figure 4).

**Table 6:** Comparison of antibody titers between the three vaccination groups at each post-vaccination-sampling interval.

**Sampling interval months post-vaccination (mpv)**	**Group 1**				**Group 2**				**Group 3**			
	**Mean VNT titer (log10)**	**SD**	**95% LCI**	**95% UCI**	**Mean VNT titer (log10)**	**SD**	**95% LCI**	**95% UCI**	**Mean VNT titer (log10)**	**SD**	**95% LCI**	**95% UCI**
1.5	1.88	0.29	1.75	2.01	1.91	0.30	1.75	2.07	1.95	0.51	1.14	2.76
3	1.64	0.19	1.54	1.74	2.33	0.29	2.17	2.49	1.78	0.10	1.63	1.93
4	1.32	0.15	1.23	1.41	1.56	0.40	1.29	1.83	1.48	0.30	1.02	1.94
5	1.45	0.16	1.36	1.54	1.79	0.21	1.65	1.93	1.58	0.05	1.50	1.66
6	1.86	0.29	1.68	2.04	1.85	0.29	1.65	2.05	1.65	0.26	1.23	2.07
7	1.38	0.16	1.25	1.51	2.72	0.47	2.23	3.21	2.93	0.51	2.13	3.73
8	1.59	0.20	1.42	1.76	2.55	0.27	2.26	2.84	2.55	0.40	1.91	3.19
9	1.49	0.09	1.41	1.57	2.57	0.22	2.34	2.80	2.58	0.34	2.04	3.12
10	1.44	0.05	1.37	1.51	2.23	0.06	2.09	2.37	2.65	0.07	2.01	3.29
11	1.52	0.08	1.51	1.53	2.23	0.06	2.09	2.37	2.60	0.00	2.60	2.60
12	1.54	0.05	1.47	1.61	2.27	0.06	2.13	2.41	2.75	0.21	0.85	4.65

*VNT, virus neutralization test; LCI, lower confidence interval; SD, standard deviation; UCI, upper confidence interval*.

## Discussion

Although mortality rates from FMDV infections are quite low (16, 44), the disease has a high public and political importance, owing to the economic strain exerted on a country facing an epidemic (45). Livestock and livestock products trade embargoes imposed on a country facing an outbreak have farther reaching financial implications than the disease control strategies employed to mitigate possible outbreaks (46). Eradication of the disease in endemic areas such as SA is impossible because of the continuous threat of livestock infection posed by the permanently FMDV-infected buffalo herds in the KNP and adjacent trans-frontier (10). Therefore, control strategies rely heavily on the preparation of efficacious vaccines which, when applied in a way that is informed by scientific data, could assist in reducing rates of outbreaks (47). This could in turn reduce the rate of trade embargoes on livestock and livestock products imposed on SA because of recurring outbreaks.

Intratypic variations within FMDV strains, characterized by a complete lack of or partial cross protection among heterologous strains, presents a challenge of successful vaccination campaigns (48). These variations are prominent within the SAT serotypes in SADC compared to the Euro-Asian serotypes (7). It is thus desirable that virus strains selected for FMD vaccine preparation should offer a broad antigenic cross protection with local heterologous field strains. This is one of the reasons why prophylactic FMD vaccines are commonly prepared as multivalent vaccines containing up to seven virus strains, as it cannot be predicted which serotypes/topotypes will be responsible for the next outbreak (11, 31).

Five strains representing the three SAT serotypes circulating in sub-Saharan Africa were previously selected for preparation of a multivalent vaccine for prophylactic and emergency vaccination campaigns. It is recommended that the efficacy of FMD vaccines be shown through animal challenge studies where vaccine efficacy, indicated by a reduction of clinical expression post-challenge, should be >75% compared to unvaccinated controls (38). The aim of our study was to demonstrate the efficacy of five local SAT type strains combined with the Montanide™ ISA 206 VG DOE adjuvant in Nguni cattle. Thus, potency, onset of antigen specific humoral response, and safety of individual strains as monovalent vaccines were evaluated. Subsequently, the duration of humoral immunity and clinical protection were evaluated over 12 months after formulation of five strains as a multivalent vaccine, to mimic field application of the vaccine.

The QC-tests conducted on all formulated vaccines showed that the vaccine formulation process did not result in the introduction of undesirable contaminating agents and antigen losses. Subsequently, administration of the highest vaccine dose 2 ml used to evaluate the five monovalent vaccinations safety did not cause adverse skin reactions at the injection sites or febrile condition post-vaccination and was thus considered safe. The onset of antigen specific humoral immunity after vaccination with 2 ml monovalent vaccine dose (indicated by antibody titers ≥2.0 log10) was observed between 7 and 14 dpv. Owing to reports that induction of neutralizing antibodies is normally considered as an important indicator of protective immunity against FMDV (49), these antibody titers were considered indicative of a good immunological response.

Protection mediated by full-dose (2 ml) vaccination across all five monovalent vaccines was full, as revealed by the absence of generalized lesions. However, a dose-dependent reduction in protection (animals showing podal lesion on at least one foot) was observed, with the exception of SAT 1B vaccine (formulated with BOT 1/06/1 strain), which elicited full clinical protection with a 0.125-ml vaccine dose. The rapid onset (7 dpv) of humoral immunity induced following vaccination with 2 ml of the two SAT 2 monovalent vaccines was attributed to higher antigen payloads (6 μg/ml) used during vaccine preparation vs. the 3 μg/ml used for SAT 1 and 3 monovalent vaccines. This response will be beneficial during outbreak emergencies as SAT 2 viruses are responsible for most outbreaks experienced in SA (50). Suffice it to mention that similar antibody titers were achieved with SAT 1B (BOT 1/06/1) vaccine, albeit at half (3 μg/ml) the antigen payload.

Foot and mouth disease vaccines are classified as either “standard” or “higher” potency vaccines (38). According to this classification, standard potency vaccines are those formulated to contain antigen concentrations resulting in the minimum potency level of 3 PD50 and thus suitable for prophylactic vaccinations. In addition, vaccination of naïve animals for control of outbreaks requires preparation of vaccines with higher potency (≥6 PD50). The potency evaluation of the five monovalent vaccines revealed that 3–6 μg of BEI inactivated SAT antigens give rise to potency values higher than the standard 3 PD50 (between 4 and 32 PD50) and rapid onset of immunity. Thus, a full dose could be used successfully for both prophylactic and emergency vaccinations, without augmentation of the antigen payloads.

An additional benefit of using higher-potency vaccines is that they overcome challenges of antigenic variations within virus strains of the same serotype and could thus protect against clinical FMD in the event of a poor vaccine match with the circulating field strain (51, 52). It is therefore anticipated that the multivalent vaccine formulated with the five strains could potentially provide adequate protection against heterologous field strains, especially during outbreak emergencies, when *in vitro* vaccine matching data are not available. Lazarus et al. (53) reported efficacy of this multivalent vaccine in goats post-SAT 1 heterologous challenge.

The humoral immunity data post-vaccination with the multivalent vaccine revealed that a single 2 ml dose elicited antibody titers ≥1.8 log10 from 1.5 mpv coinciding with full clinical protection of naïve cattle post-SAT 2 homologous challenge. Booster vaccination at 1.5 mpv resulted in a further increase of antibody titers up to 3 mpv and full clinical protection. However, without this booster vaccination, a decline in antibody titers (<1.6 log10, negative cut-off) and a reduction in clinical protection (75%) were observed. The benefit (clinical protection) of the 1.5 mpv booster vaccination was not experienced when animals were challenged 6 months after the primary vaccination. Similar to the animals which were not boosted, 60% (*n* = 3/5) clinical protection was recorded in the boosted animal group, even though antibody titers greater than the baseline line (day 0) response were recorded. Thus, a correlation between the antibodies before challenge and clinical protection post-challenge could not be drawn.

Administration of the 6 mpv booster vaccination, either as a first or second booster of the primary vaccination, amplified the antibody titers (≥2 log10) from 7 mpv onwards, exceeding those observed after 1.5 mpv booster vaccination. At the time of primary vaccination several cattle succumbed to acute, hemorrhagic fibrinonecrotic pneumonia, which was attributed to environmental stress. Several animals were rigorously treated with antibiotics and recovered. It is therefore suspected that the low humoral immune responses seen after the 1.5 mpv booster were due to inadequate immune responses in the sick animals. Contrary to this, at 6 mpv booster vaccination all experimental animals had fully recovered from the illness and had adjusted to the environmental conditions in their holding pens. Therefore, 100% clinical protection post-challenge at 9 and 12 mpv was observed, although the number of animals challenged had reduced due to the death of several animals. Improvement in clinical protection was also seen in animals that were never boosted post-primary vaccination even though their antibody titers were ≤ 1.6 log10. This unanticipated clinical protection could be attributed to a combination of several factors including the role of high-potency vaccines (54), cell-mediated immunity (55, 56), and the contribution of the DOE adjuvant used for vaccine preparation (27).

Double oil emulsion Montanide™ ISA 206 VG adjuvanted FMD vaccines induce longer-lasting immunity compared to aluminum hydroxide adjuvanted vaccines (27–29), especially if frequent booster vaccinations are applied (26). This is because the vaccine is formulated such that the antigen is readily available in the first continuous aqueous phase of the emulsion for induction of short-term immunity upon injection, and subsequently, long-term immunity is triggered by the secondary aqueous phase encapsulated within the oil droplets of the first aqueous phase (33).

One of this study's limitations is that it evaluated the efficacy of the vaccine using protection against generalized FMDV infection, without assessment of viral shedding and the development of a carrier state in vaccinated animals. This approach assumed a probability of protection wherein 75% protection was considered sufficient to support the application of the vaccines in susceptible populations (19). Nonetheless, this does not detract from the overall conclusion that can be drawn from the results. Previous studies in cattle have shown that although a virus could be isolated up to 57 (57) or 98 (58) dpc from vaccinated and challenged animals, the virus was not transmitted to naïve cattle following direct contact with these carrier animals. Additionally, live virus could not be isolated post-challenge from the cattle vaccinated 7 days or longer with a commercial, DOE vaccine using inactivated purified O Manisa strain of FMDV with a 3 PD50 (57). It can therefore be reasonably assumed that vaccination would have a discernible effect preventing transmission of FMDV by reducing the manifestation of clinical disease, even if sterilizing immunity is not achieved.

Owing to cost considerations, the study evaluated the duration of immunity against SAT 2 challenge, as this serotype is the cause of most outbreaks in SA (50). Thus, it cannot be concluded empirically that the multivalent vaccine could protect against SAT 1 and 3 outbreaks in cattle. However, a recent study conducted in goats using the same multivalent vaccine used in the current study found that none of the animals vaccinated with a full one-third and one-sixth dose had evidence of viral RNA detected in oropharyngeal, nasal, and rectal swab specimens from 0 to 6 dpc (53). In a different study, the efficacy of a thermo-stabilized SAT 2 vaccine showed that protection mediated by the DOE Montanide ISA 206 adjuvant elicited higher SAT 2 neutralizing antibody titers and systemic IFN-γ responses at 14 and 28 dpc (59).

Based on these data, we consider the five candidate vaccine strains suitable for successful local prophylactic vaccinations and elsewhere in the world where antigenically related SAT serotypes occur. Owing to high potency, we anticipate that the application of a 2 ml multivalent vaccine could also be utilized successfully during outbreaks emergencies. The ANOVA between the three multivalent vaccination regimes revealed an insignificant (*p* > 0.05) immunological difference between application of one or two booster vaccinations. However, we recommend the administration of a 2 ml primary vaccination dose to 6-month-old naïve cattle followed by booster vaccinations at 1.5 and 6 mpv, owing to the decline in both humoral response and clinical protection recorded 3 months after primary vaccination in animals which were not boosted 1.5 mpv. We therefore anticipated that this vaccination regimen could potentially maintain a 100% herd protection over a 12-month period.

## Data Availability Statement

The original contributions presented in the study are included in the article/Supplementary Material, further inquiries can be directed to the corresponding author/s.

## Ethics Statement

The animal study was reviewed and approved by 1. Onderstepoort Veterinary Institute—Animal Ethics Committee, 2. South African Department of Agriculture, Land Reform and Rural Development, Section 20 of the Animal Disease Act 35 of 1984, Directorate of Animal Health, and 3. Sefako Makgatho University of Health Sciences Research Committee.

## Author Contributions

FP: conceptualization, methodology, investigation, data curation, and write-up (original draft). MS: statistical analysis. PB and PM: methodology and data curation. NV: technical supervision and data curation. JO and MS: academic supervision. LH: project administration, supervision, and data curation. All authors contributed to the article and approved the submitted version.

## Funding

The authors would like to acknowledge funding from the South African National Treasury for the overarching funding of the FMD Vaccine Factory upgrade project, awarded to the Agricultural Research Council.

## Conflict of Interest

PB and NV were employed by company Private Consultants, Boxmeer. The remaining authors declare that the research was conducted in the absence of any commercial or financial relationships that could be construed as a potential conflict of interest.

## Publisher's Note

All claims expressed in this article are solely those of the authors and do not necessarily represent those of their affiliated organizations, or those of the publisher, the editors and the reviewers. Any product that may be evaluated in this article, or claim that may be made by its manufacturer, is not guaranteed or endorsed by the publisher.
