# Supplementary material for: Evaluation of Potency and Duration of Immunity Elicited by a Multivalent FMD Vaccine for Use in South Africa
Source: Front Vet Sci. 2021 Dec 15;8:750223. doi: 10.3389/fvets.2021.750223 (PMC8714748; doi:10.3389/fvets.2021.750223)
Supplement: Supplementary file 1 [file Data_Sheet_1.PDF]

SUPPLIMENTARY DATA

EVALUATION OF POTENCY AND DURATION OF IMMUNITY ELICITED BY A MULTIVALENT FMD VACCINE FOR USE IN SOUTH AFRICA

1. Virus inactivation and clarification

Pro-analysis Chloroform (Merck) was added to the virus cultures to a final concentration of 0.3% and the virus harvests clarified by centrifugation at 2000 rpm for 10 minutes in a Sorvall RC 3C Plus refrigerated centrifuge with H6000A rotor. Inactivation was conducted according to Bahnemann (1990), for 24 hours at 35° C using two doses of 2.5 mM Binary Ethyleneimine (BEI) each, at the beginning of inactivation (0 hours) and after 4 hours when the culture was transferred to the second inactivation vessels. Samples were collected before inactivation and followed by every hour up to six hours, and at the end of inactivation (24 hours.) Collected samples were subjected to the virus titration test described in 2.1 to evaluate if the viruses inactivation kinetics complies with the statutory regulations described in the OIE manual of Diagnostic Tests and vaccines for terrestrial Animals, INATIVATION (19).

Samples collected at the end of inactivation were also subjected to the sterility and 146 S particle quality control (QC) tests.

2. In-process QC tests

2.1 Virus titration test (after virus inactivation process)

BHK-21 cell suspension of a concentration of 1 x 10^5 c/ml was used as a diluent of virus samples collected during inactivation. Each inactivation sample was diluted in a series of 10^1 up to 10^9 (10-fold) dilutions and each dilution per sample cultured in eight wells of 96-well flat-bottomed Nunc plates. Based on the CPE observed per dilution series in 96 wells plates after incubation at 37 °C, in an atmosphere of 5% CO_2 for 72 hours (± 4 hours), virus titres per inactivation sample were established. The titres were expressed as the reciprocal of the highest virus dilution that produced CPE in 50% of the inoculated cells.

The Reed and Muench (1938) method of end point assessment was used to calculate the titres per inactivation sample which were expressed as the 50% tissue culture infectious dose per millilitre of virus sample (TCID50/ml) to plot the logarithmic drop in virus infectivity against the total inactivation time. Virus titers were used to show logarithmic drop in virus infectivity against the total inactivation time (Table 1).

2.2 Antigen innocuity test (after concentration and purification process)

The absence of residual live virus in concentrated antigens was confirmed by two consecutive passages of concentrated and purified antigen samples representing 200 vaccine doses of each vaccine strain in 850 cm roller bottles (Coning) of BHK 21 clone 13 monolayer cell cultures, (according to the method in the OIE Manual of Diagnostic Tests and Vaccines (19). The absence of residual live virus was indicated by absence of CPE in the second passage of the antigens, after 48 hours incubation at 37°C.

2.3 Sterility test as described previously (19, 36)

The virus harvest, inactivation, concentrated antigen, and the vaccine samples were tested for freedom from undesirable microbial contaminants following procedures outlined in the European Pharmacopoeia, 2006 Monograph. Briefly, samples were inoculated onto tryptose soya, thioglycolate and nutrient broths. In addition, the same samples were cultured onto blood tryptose and brain infusion agar plates. All inoculated testing materials were incubated aerobically and anaerobically in an atmosphere of 5 % carbon dioxide at ca. 37 °C for 14 days. The samples were considered to have passed the test if the agar plates did not show any microbial colonies and the broth media did not turn turbid when observed with the naked eye.

2.4 146 S particle test as described previously (37-38)

The individual 146 S particle concentration of the five after cultivation as well as the concentration and purification process was determined using sucrose gradients ultracentrifugation as described previously (37-38), using the Teledyne ISCO fractionation system connected to ultraviolet detector, at 254 nm. Briefly, sucrose gradients were prepared from 10 and 30% sucrose solutions and the samples to be tested were loaded onto them. The gradients were ultracentrifuged at 45 000 rpm for 40 minutes at 4 (± 0.5) °C using the Optima XPN ultracentrifuge with a SW 55 Ti swinging bucket rotor (Beckman Coulter Life Sciences, South Africa).

Using a phenol red-stained 50% sucrose solution and a peristaltic pump, gradients with samples were pumped through a LKB UVIcord SII ultraviolet detector connected to a recorder set at an absorbency of 254 nm. The surface area under the peak was measured and the amount of the 146 S particle in the peak calculated using the peak integration Clarity™ advanced chromatography software for Windows (DataApex, The Czech Republic) and expressed in µg/ml.

2.4.1 Extraction of antigen from formulated vaccines for 146 S particle test.

Antigens were precipitated from the vaccine by addition of equal volumes of chloroform and 1M KH_2PO_4 solution, to final concentration of 0.1 %. The mixtures were centrifuged at 1000 rpms 25 °C for 10 minutes and antigens collected as supernatants for 146 S particle test.

3. Results

The inactivation kinetics, 146 S particle, sterility and innocuity tests results of the five antigen samples are summarized in Table 1. Samples collected during inactivation of the viruses indicated that the five antigens fulfilled the inactivation kinetics requirements stipulated in OIE manual of Diagnostic Tests and vaccines for terrestrial Animals (19). Additionally, all samples collected for sterility test passed. No residual live virus was obtained during innocuity testing of the concentrated antigens.

Table 1: A summary QC test results during antigen production for vaccine formulation.

**TEST**	**Description of test conducted**	**compulsory requirements for the tests**	**RESULTS PER STRAIN**				
			SAT 1A	SAT 1B	SAT 2A	SAT 2B	SAT 3
INACTIVATION KINETICS	Virus inactivation kinetics (the rate of viral RNA inactivation):	**Correlation coefficient R= < -0.90**	-0.997	-0.978	-0.996	-0.978	-0.90
	Calculated safety titer reached at two-thirds of inactivation time (16 hours for a 24 hours inactivation time)	**Safety titre**<br>**-7.0 log 10**	-10.03	-13.53	-16.15	-13.58	-18.00
	Time it took to reach safety titer:	**Time ≤ 16 hours (h)**	13.25 h	10.81 h	09.7 h	10.78 h	09.40 h
146 S PARTICLE	Concentration **(μg/ml) of** 24 hours inactivation sample	**≥ 0.5 μg/ml**	2.41	2.20	0.60	2.30	0.90
STERILITY	Absence of microbial contaminants	**Negative**	Pass	Pass	Pass	Pass	Pass
INNOCUITY	Absence of residual live virus before vaccine formulation	**Negative**	Pass	Pass	Pass	Pass	Pass

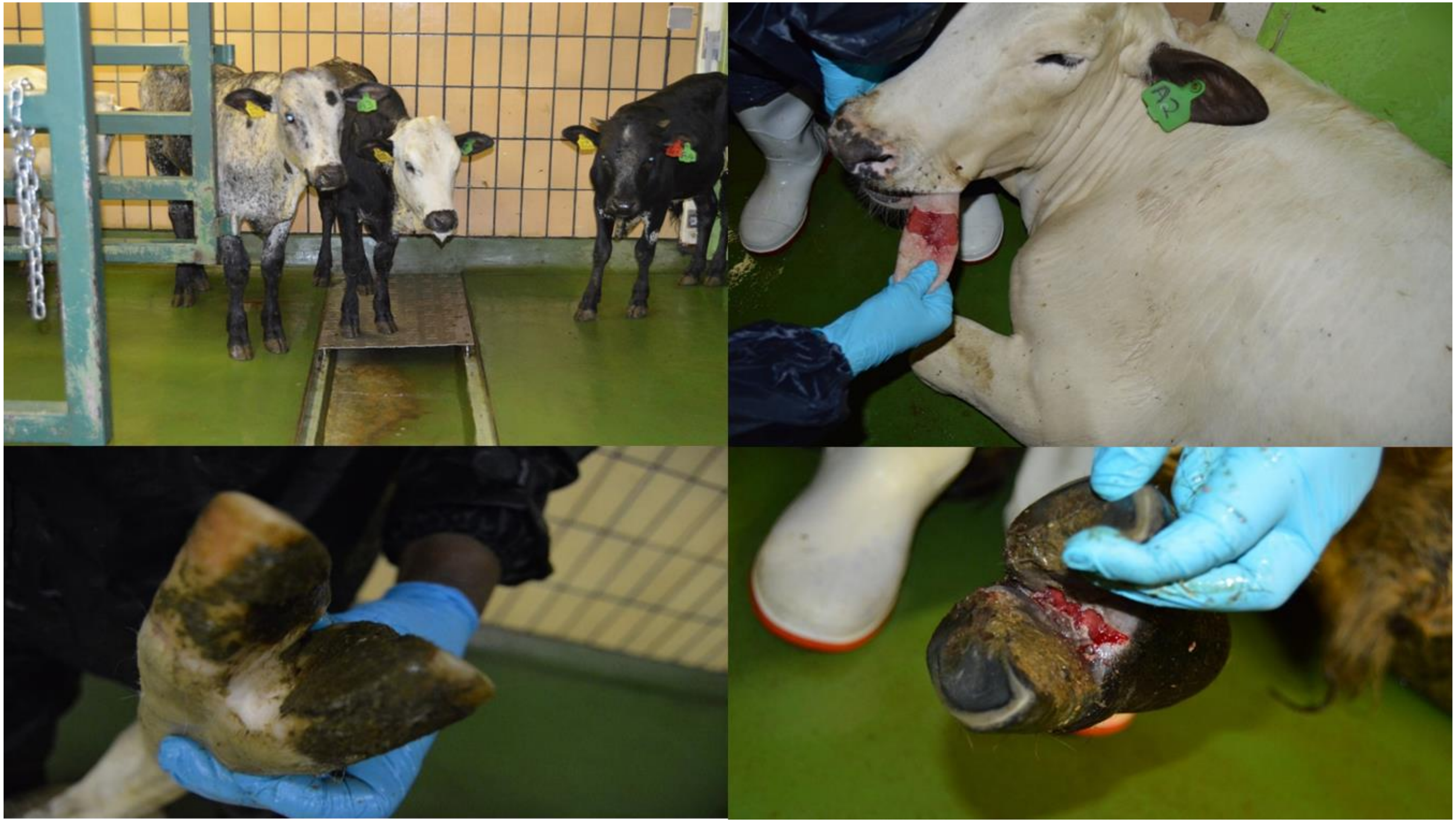

Figure 1: Clinical signs caused by challenge virus: Typical FMDV tongue lesions, foamy salivation associated with FMDV tongue lesion in vaccinated, absence of hoof (foot) lesions in a negative animals (unchallenged)
